# Supplementary material for: Mobile phone carrying locations and risk perception of men: A cross-sectional study
Source: PLoS One. 2022 Jun 7;17(6):e0269457. doi: 10.1371/journal.pone.0269457 (PMC9173639; doi:10.1371/journal.pone.0269457)
Supplement: Attachment — Submitted filename: Author_Response_PONE-D-21-18441.docx [file pone.0269457.s001.docx]

**Journal Requirements:**

When submitting your revision, we need you to address these additional requirements.

1. Please ensure that your manuscript meets PLOS ONE's style requirements, including those for file naming. The PLOS ONE style templates can be found at

<https://journals.plos.org/plosone/s/file?id=wjVg/PLOSOne_formatting_sample_main_body.pdf> and <https://journals.plos.org/plosone/s/file?id=ba62/PLOSOne_formatting_sample_title_authors_affiliations.pdf>

**AR: The current version is formatted according to the formatting requirements**

2. Thank you for stating the following in the Acknowledgments Section of your manuscript:

“This research project is supported by the Centre for Population Health Research on Electromagnetic Energy (PRESEE), School of Public Health and Preventive Medicine, Monash University. The centre is funded by a grant from the National Health and Medical Research Council, Australia (APP 1060205).”

We note that you have provided additional information within the Acknowledgements Section that is not currently declared in your Funding Statement. Please note that funding information should not appear in the Acknowledgments section or other areas of your manuscript. We will only publish funding information present in the Funding Statement section of the online submission form.

Please remove any funding-related text from the manuscript and let us know how you would like to update your Funding Statement. Currently, your Funding Statement reads as follows:

“This research project is supported by the Centre for Population Health Research on Electromagnetic Energy (PRESEE), School of Public Health and Preventive Medicine, Monash University. The centre is funded by a grant from the National Health and Medical Research Council, Australia (APP 1060205).

The funders had no role in study design, data collection and analysis, decision to publish, or preparation of the manuscript.”

Please include your amended statements within your cover letter; we will change the online submission form on your behalf.

**AR: We have now deleted the funding in the acknowledgement section of the main manuscript. Please insert the following information regarding funding on our behalf. *“This research project was supported by the Centre for Population Health Research on Electromagnetic Energy (PRESEE), School of Public Health and Preventive Medicine, Monash University. The centre was funded by a grant from the National Health and Medical Research Council, Australia (APP 1060205). The funder had no role in the study design, data collection and analysis, decision to publish, or preparation of the manuscript.”***

3. We note that you have included the phrase “data not shown” in your manuscript. Unfortunately, this does not meet our data sharing requirements. PLOS does not permit references to inaccessible data. We require that authors provide all relevant data within the paper, Supporting Information files, or in an acceptable, public repository. Please add a citation to support this phrase or upload the data that corresponds with these findings to a stable repository (such as Figshare or Dryad) and provide and URLs, DOIs, or accession numbers that may be used to access these data. Or, if the data are not a core part of the research being presented in your study, we ask that you remove the phrase that refers to these data.

**AR: Thank you. We have now removed the phrase as the data related to these were not a core part of our study.**

4. Please ensure that you refer to Figure 3 in your text as, if accepted, production will need this reference to link the reader to the figure.

**AR: Figure 3 is now cited in text**

**Reviewers' comments:
Reviewer's Responses to Questions**

**Comments to the Author**
1. Is the manuscript technically sound, and do the data support the conclusions?
The manuscript must describe a technically sound piece of scientific research with data that supports the conclusions. Experiments must have been conducted rigorously, with appropriate controls, replication, and sample sizes. The conclusions must be drawn appropriately based on the data presented.

Reviewer #1: Yes

Reviewer #2: Yes

2. Has the statistical analysis been performed appropriately and rigorously?

Reviewer #1: Yes

Reviewer #2: Yes

3. Have the authors made all data underlying the findings in their manuscript fully available?
The [PLOS Data policy](http://www.plosone.org/static/policies.action#sharing) requires authors to make all data underlying the findings described in their manuscript fully available without restriction, with rare exception (please refer to the Data Availability Statement in the manuscript PDF file). The data should be provided as part of the manuscript or its supporting information, or deposited to a public repository. For example, in addition to summary statistics, the data points behind means, medians and variance measures should be available. If there are restrictions on publicly sharing data—e.g. participant privacy or use of data from a third party—those must be specified.

Reviewer #1: No

Reviewer #2: Yes

**AR: Thank you. We have now removed the phrase as the data related to these were not a core part of our study.**

4. Is the manuscript presented in an intelligible fashion and written in standard English?
PLOS ONE does not copyedit accepted manuscripts, so the language in submitted articles must be clear, correct, and unambiguous. Any typographical or grammatical errors should be corrected at revision, so please note any specific errors here.

Reviewer #1: Yes

Reviewer #2: Yes

5. **Review Comments to the Author**
**Reviewer #1: Summary:**
In this article, the authors explore the relationship between mobile phone carrying and the risk perception of RF-EMF exposure due to carrying close to the body. The study is done by asking 356 participants (men) a self questionnaire for their mobile phone use. The authors describe statistics of overall usage when they use mobile phones indoor, outdoor, and when receiving calls. Finally, the authors analyze the survey data with the risk perception. The analysis is done mainly using correlation and linear regression models. They found that men generally carry their phone close to reproductive organs (trouser front pocket). And men who kept their phones away from their body were less likely to perceive health risks from their behaviour.
**Overall feedback:**
Overall, the dataset and descriptive statistics are well described in the article. However, the introduction part can be improved where authors can give solid statements of why readers should care about the survey. Moreover, there is still a lack of discussion in the article. The authors can improve and solidify the overall message of the paper.

**AR: Thank you very much. In the current version of the manuscript, we have revised the introduction and the changes we made are reflected in the tracked changes**

**Comments:**
Introduction. The authors describe overall research in the introduction well but it is still hard to make a clear statement of why there is a need to analyze the survey from the reader's perspective. There is still a lack of a gap in the first paragraph. It would be better if authors can provide a more solid paragraph of why risk perception is needed in this study. I think it would be good for readers to have a one paragraph about health risks of carrying mobile phones, why risk perception is needed, and why surveys are needed.

**AR: We have now revised paragraph 1 and paragraph 2 of the introduction and we have added the following, “ *According to the International Telecommunication Union, 85% of the global population have access to telecommunication networks and 57% to internet services (1).*** ***In addition to making and receiving calls, digital technologies available in smartphones enabled access to several services such as voice data (e.g., making or receiving a call), and video data (messenger, WhatsApp, YouTube, etc.) (2). Mobile phones emit and receive the data via radiofrequency electromagnetic fields (RF-EMF).***

***Mobile phone usage related RF-EMF exposure and likely human health effects have long been of concern internationally (3, 4).*** ***RF-EMF exposure from mobile phones may induce some heating effect and/or a sensation of warmth, depending upon a range of factors, including the distance between the mobile phone and part of the body exposed during active use (5, 6). A recent systematic review reported that studies speculated that scrotal overheating might affect male fertility (7).*** ***In another observational study, men who carried their mobile phones in their hip pockets or on their belts had lower sperm motility than men who did not carry a mobile phone or who carried their mobile phone elsewhere on the body (8). In view of this, understanding of people’s habits of placing/carrying a mobile phone becomes important.”***

Methods. The authors describe the dataset well. I do not see the statement of data availability. It would be good to make an anonymous survey available.

**AR: Thank you. Data would be made available once other upcoming analysis is completed**

Results and discussions. The author explains why risk perception for men who carry phones close contact is higher. It would be good to explain the result more why they might have higher risk perception. In the discussion, it feels more like describing the data but not much insight with the collected data. Overall, I think the writing can be much stronger if authors provide some more evidence or further discussion. For example, why men who carry phones near their body are more likely to perceive more health risks? And here, if they perceive more risk, why do they still carry their phone near the pocket?

**AR:** **This was a cross-sectional survey with limitations inherent to the design. It was not possible to tell which came first –exposure or outcome?** **In the case of carrying mobile phone close to body parts and risk perception, we suspect that if one carried his mobile phone close to body parts, he might be prompted by being inquired about it and hence reminded of negative perceived effects. Furthermore, risk perception may not be translated to action such as not carrying mobile phones close to body parts. We have revised the discussion section and it now reads as, “*****If one carries his mobile phone close to body parts, he might be prompted by being inquired about possible negative effects and may rate higher levels of risk perception.”***

**Reviewer #2:**

In order to publish a research article in a journal, a manuscript should describe a technically sound piece of scientific research with data that supports the conclusions. Experiments must have been conducted rigorously, with appropriate controls, replication, and sample sizes. From this point of view, the article appears to be a survey study instead of a technically sound piece of scientific research. I believe that the article should not be classified as a research article.

**AR: We respectfully disagree with this comment. Although we agree that experimental studies are considered gold standard designs, cross-sectional studies are also valid study designs widely employed in Epidemiology, clinical, social and public health research.**
