# Supplementary material for: Mobile phone carrying locations and risk perception of men: A cross-sectional study
Source: PLoS One. 2022 Jun 7;17(6):e0269457. doi: 10.1371/journal.pone.0269457 (PMC9173639; doi:10.1371/journal.pone.0269457)
Supplement: Attachment — Submitted filename: PONE-D-21-18441R1_Authors_response_R2.docx [file pone.0269457.s002.docx]

**Reviewers' comments:
Reviewer's Responses to Questions**

**Comments to the Author**

1. If the authors have adequately addressed your comments raised in a previous round of review and you feel that this manuscript is now acceptable for publication, you may indicate that here to bypass the “Comments to the Author” section, enter your conflict of interest statement in the “Confidential to Editor” section, and submit your "Accept" recommendation.

Reviewer #3: (No Response)

Reviewer #4: All comments have been addressed

2. Is the manuscript technically sound, and do the data support the conclusions?

   The manuscript must describe a technically sound piece of scientific research with data that supports the conclusions. Experiments must have been conducted rigorously, with appropriate controls, replication, and sample sizes. The conclusions must be drawn appropriately based on the data presented.

Reviewer #3: Yes

Reviewer #4: Yes

3. Has the statistical analysis been performed appropriately and rigorously?

Reviewer #3: Yes

Reviewer #4: Yes

4. Have the authors made all data underlying the findings in their manuscript fully available?

   The [PLOS Data policy](http://www.plosone.org/static/policies.action#sharing) requires authors to make all data underlying the findings described in their manuscript fully available without restriction, with rare exception (please refer to the Data Availability Statement in the manuscript PDF file). The data should be provided as part of the manuscript or its supporting information, or deposited to a public repository. For example, in addition to summary statistics, the data points behind means, medians and variance measures should be available. If there are restrictions on publicly sharing data—e.g. participant privacy or use of data from a third party—those must be specified.

Reviewer #3: Yes

Reviewer #4: Yes

5. Is the manuscript presented in an intelligible fashion and written in standard English?

   PLOS ONE does not copyedit accepted manuscripts, so the language in submitted articles must be clear, correct, and unambiguous. Any typographical or grammatical errors should be corrected at revision, so please note any specific errors here.

Reviewer #3: Yes

Reviewer #4: Yes

6. **Review Comments to the Author**

   Please use the space provided to explain your answers to the questions above. You may also include additional comments for the author, including concerns about dual publication, research ethics, or publication ethics. (Please upload your review as an attachment if it exceeds 20,000 characters)

**Reviewer #3:**

The authors present a cross-sectional study investigating a novel and interesting research question. In fact, due to their widespread use in almost any society, studies addressing research questions related to mobile phones and the resulting RF-EMF exposure are of considerable interest for public health. In my view, the authors satisfactorily addressed the comments by Reviewer 1 in the first round of reviews, even though of course this primarily needs to be evaluated by Reviewer 1. Moreover, I agree with their reply to Reviewer 2; in my opinion, there is no doubt that an observational study should be classified as a research article. Nevertheless, I would recommend that the authors take into account the following questions and remarks before the manuscript is released for publication.

**AR: Thank you**
Abstract: I would suggest to add the sampling strategy (convenience sampling) to the abstract. Ideally, apart from the number of participants also the response rate should be provided but I assume that this rate cannot be calculated because of the sampling method (making it vice versa even more important to mention the sampling strategy).
I would also suggest to add “by means of linear regression models” to the sentence “Data were analysed to examine if…”.

Could you, if the word count allows, provide specific numbers (regression coefficients, p-values) to the statement “Men who kept their handset away from the body parts […] were less likely to perceive health risks form their behaviour”?

**AR: We have added the analysis model in the abstract and it reads as, “*****Data were analysed using linear regression models to examine if risk perception differed by mobile phone carrying location”*. In addition, we have included information about the levels of significance. We have also revised the limitation section of the discussion to reflect the sampling limitations (see the last paragraph of discussion section).**

Introduction:
Page 4, second sentence: Can you add the country where this study (women aged 15-40 years) was performed? I think this information may be of interest since in the previous sentence you mention cultural differences as to where individuals carry their phone.

**AR: We have now added information that the study was from Australia**

Methods:
Page 5, first paragraph: As mentioned above, it would be interesting to have a response rate. Do you happen to have any information on how many individuals you approached? In any case, I think the strategy of using a convenience sampling should be mentioned as a limitation of the study in the discussion section.

**AR: We have now added this to the limitation section and it reads as, “*****Participants were recruited via advertisements and may be pre-selected based on their access to the invitations and voluntary expression of their interest for participation and hence may not be representative of the source population.***”

Page 5, second paragraph: Could you specify “handset carrying location information” (for instance, for which specific locations did you ask?) or provide the full questionnaire in the supplement?
**AR: We have revised the paragraph and added the following information: “*Mobile handset carrying locations of men were assessed by asking them to rate, on a Likert-scale (1 = never; 2 = rarely, 3 = occasionally, 4 = often, 5 = very often), how commonly they carry it with their hand, keep it on a table, in a bag, or in the pocket of their T-shirt, Jacket or Trousers (front or back).”***

Results:
Page 9, second paragraph. The statement “If the mobile phone was not in use, 34.7% placed it in close contact with the body…” comes a little bit as a surprise when in the sentence before you state that when not in use, 54% placed it on a table/desk and 46% in the trouser pocket. Do the 34.7% refer to not in use outdoors? Can you perhaps clarify this?

**AR: We have revised the sentence and it now reads as, *“Of the men who keep their mobile phone in close contact to their body parts, 46.0% keep it in the trouser front pocket.”***

In my view, the high proportion (78.7%) of participants owning a mobile for more than 20 years appears pretty high? As far as I am aware, even though mobile phones became available in the 1990s, their widespread use did not kick in before the early 2000s (although I am not sure how the situation is in Australia), indicating that the majority of your participants seem to be (very) early adopters. Do you think this may point towards some form of selection bias in the sense that you recruited a relatively tech-savvy sample (which may differ with respect to phone use or risk perception compared to the general population)? In my view, it would be worthwhile to elaborate on this issue in a few sentences in the discussion part.

**AR: Thank you for the comment. The high prevalence of use might be unique case to Australia. For example, in a study conducted over a decade ago 68% of children aged 10-11 years (in 2012), reported having owned or used a mobile phone.**

What I also see as quite surprising is your finding that men carrying their phone close to the body reported higher risk perception levels than those placing it in a bag or table. Would you not expect that individuals more concerned about potential health effects carry their phone farther away from their body? I think this may also be an interesting question to be addressed in the discussion.

**AR: We agree that one can expect risk may impact behaviour to some extent such as not to carry the handset close to reproductive organs if one perceived risk. However, being a cross-sectional study, it may be that one was prompted about the possible risks and rated a higher risk if already carrying the phone in close proximity to the body. This was discussed in the earlier version of the manuscript (discussion section: paragraph 2), and reads as,** “***There is some evidence that perception of risk may impact behaviour to some extent such as whether or not to carry the handset close to reproductive organs (8). Men who kept their mobile phone away from body-parts while it was not in use were half as likely to perceive health risks from the specific behaviour. If one carries his mobile phone close to body parts, he might be prompted by being inquired about possible negative effects and may rate higher levels of risk perception.”***

Discussion:
Page 13, first paragraph: “For example, up to 64% of men…” Can you add a reference for this study?

**AR: We have now cited a reference**

In general, I would recommend that in the first paragraph you highlight the most important findings of your study (including those on risk perception levels) and then start with the comparison to other studies in the second paragraph.

**AR: We have revised this and divided the paragraph accordingly. We have added the following to the discussion (parag 1), “*Men who carried their handset in close contact with their body, perceived higher risks from RF-EMF exposure compared to those who kept it away from the body. However, they were less likely to perceive health risks from their mobile phone handset carrying behaviour.”***

Page 15, first paragraph: I think the potential of selection bias is a more important aspect here then the question of the generalisability of your study to other parts of Australia. As mentioned above, the use of a convenience sample and the potential of selection bias, e.g. with respect to attraction to use of technical devices in general and mobile phones in particular, and potential implications for the interpretation of your numbers, should receive some attention. If I am not mistaken, there is some evidence from other studies on mobile phone use that individuals with higher levels of usage tend to have a higher interest to participate in such studies than non-regular users. On the other hand, it may also seem plausible that people with higher levels of concern regarding health effects are more prone to join such as study as yours (which may lead to an overestimation of the true level of risk perception in the general population). To make a long story short, it would be great if you could add your thoughts on those aspects to the discussion part.

**AR: We have now added this to the limitation section and it reads as, “*Participants were recruited via advertisements and may be pre-selected based on their access to the invitations and voluntary expression of their interest for participation and hence may not be representative of the source population.***”

Minor comments:

Abstract, second sentence: “… and to assess relationship…” should read “… and to assess its/the relationship…” (add “its” or “the”)
**AR: Thank you. We added “the”**
In the second part of the abstract, some percentages are provided with decimal places and some without. I would recommend to harmonise this.
**AR: Corrected**
Introduction, third sentence: “enabled” should read “enable” as all other sentences in this paragraph are framed in present tense.
**AR: Corrected**
Results, first sentence: The information that 356 men participated is redundant since the sample size is already mentioned in the methods section. I would thus recommend to remove the first sentence and add the age range to the sentence describing the mean (SD) age of participants.
**AR: We have now deleted the sentence describing the mean (SD) age of participants as it is already presented in the table. Sample sized also removed from results section.**
Page 14, last sentence: Add a comma after “body”
**AR: Corrected**
Page 15, first paragraph: “eg winter” should read “e.g., winter”

**AR: Corrected**

**Reviewer #4:**

I do think that the manuscript merits to be published on PlosOne. In my opinion, Authors correctly addessed the comments of Reviewer 1. Moreover, I agree with Authors on their answer to the comment of Reviewer 2.

I have only a minor point to raise:

Table 1, Education: what does it means TAFE? On the Internet you can find some clues, e.g. :Australian Technical and Further Education (TAFE) institutes are government-owned providers of VET courses (Vocational Education and Training). Authors have to be more explicit, also with the use of a footnote that permits to the readers to understand the meaning of that acronym (without surfing the Internet!)

**AR: We have now replaced TAFE with Vocational training (diploma).**

7. PLOS authors have the option to publish the peer review history of their article ([what does this mean?](https://journals.plos.org/plosone/s/editorial-and-peer-review-process#loc-peer-review-history)). If published, this will include your full peer review and any attached files.
If you choose “no”, your identity will remain anonymous but your review may still be made public.
Do you want your identity to be public for this peer review? For information about this choice, including consent withdrawal, please see our [Privacy Policy](https://www.plos.org/privacy-policy).

Reviewer #3: Yes: Tobias Weinmann

Reviewer #4: Yes: Stefano Mattioli
